# Olanzapine as a prophylactic antiemetic for preventing postoperative nausea and vomiting after general anesthesia: A systematic review and meta-analysis

**DOI:** 10.1016/j.clinsp.2025.100645

**Published:** 2025-04-05

**Authors:** Thiago Ramos Grigio, Hans Timmerman, Angela Maria Sousa, André Paul Wolff

**Affiliations:** a Groningen Institute for Evolutionary Life Sciences, Groningen, the Netherlands; b University of Groningen, Groningen, the Netherlands; c Universidade de São Paulo, São Paulo, SP, Brazil

Dear Editor,

We appreciate the comments by Ribeiro PF and colleagues ^1^ regarding our published systematic review and meta-analysis on the prophytactic use of olanzapine for postoperative nausea and vomiting ^2^. Their insights contribute valuable discussion to the field and highlight important considerations regarding study selection, heterogeneity, and meta-analysis methodology.

We acknowledge the concerns regarding including studies with varying interventions, comparisons, and outcomes. While we recognized the heterogeneity among the studies, we considered that the small number of available randomized controlled trials warranted a comprehensive inclusion to better reflect the existing evidence. In addition, a minimum of two studies are needed to do a meta-analysis because all other synthesis techniques are less transparent and/or are less likely to be valid.^3^ Our approach aligns with the recommendations in systematic reviews where heterogeneity can be addressed through appropriate statistical models, such as random-effects meta-analysis.^4^ Furthermore, we only conducted a subgroup analysis to evaluate different doses of olanzapine because there were few studies included. Other subgroup analysis was not possible due to this limitation.

The potential overestimation of effects is a valid concern. However, we decided to include all eligible studies to provide an initial synthesis of available evidence. If more homogeneous definitions of interventions, outcomes, and time-dependent criteria were available, we would re-evaluate the inclusion criteria to improve comparability. Future research employing more standardized methodologies and incorporating time-dependent criteria will undoubtedly strengthen the conclusion on this topic.

We hope our systematic review will inspire further research with more robust designs and larger sample sizes to address the gaps and limitations highlighted in our work. We thank you for your thoughtful comments and constructive feedback.

## Declaration of competing interest

The authors declare no conflicts of interest.
